# Safe management of carotid body tumor resection without preoperative embolization: an Ecuadorian high-altitude cities experience

**DOI:** 10.1093/jscr/rjac598

**Published:** 2022-12-20

**Authors:** Paola Solis-Pazmino, Eduardo Pilatuna, Belen Tite, Mikaela García, Richard Godoy, Carla Rocha, Oscar J Ponce, Benzon Dy, Cristhian García

**Affiliations:** Instituto de la Tiroides y Enfermedades de Cabeza y Cuello (ITECC), Quito- Ecuador; Surgery Department Hospital Santa Casa de la Misericordia, Porto Alegre, Brazil; CaTaLiNa (Cancer de Tiroides en America Latina); Instituto de la Tiroides y Enfermedades de Cabeza y Cuello (ITECC), Quito- Ecuador; CaTaLiNa (Cancer de Tiroides en America Latina); Instituto de la Tiroides y Enfermedades de Cabeza y Cuello (ITECC), Quito- Ecuador; CaTaLiNa (Cancer de Tiroides en America Latina); Instituto de la Tiroides y Enfermedades de Cabeza y Cuello (ITECC), Quito- Ecuador; CaTaLiNa (Cancer de Tiroides en America Latina); Instituto de la Tiroides y Enfermedades de Cabeza y Cuello (ITECC), Quito- Ecuador; CaTaLiNa (Cancer de Tiroides en America Latina); Instituto de la Tiroides y Enfermedades de Cabeza y Cuello (ITECC), Quito- Ecuador; IpathLab: Instituto de Patología y Medicina de Laboratorio, Quito-Ecuador; Instituto de la Tiroides y Enfermedades de Cabeza y Cuello (ITECC), Quito- Ecuador; Endocrine Surgery Department, Mayo Clinic, Rochester, Minnesota, USA; Instituto de la Tiroides y Enfermedades de Cabeza y Cuello (ITECC), Quito- Ecuador

**Keywords:** carotid body tumors, carotid glomus, Shamblin classification, without preoperative embolization, pre-TAE

## Abstract

Carotid body tumors (CBTs) are a neoplasm that affects the carotid glomus. This study aims to improve the management of CBTs in Ecuador. This single-center, retrospective observational study was conducted at the Instituto de la Tiroides y Enfermedades de Cabeza y Cuello (ITECC). We included adults with CBTs, between January 2019 and August 2022. A total of 15 patients with CBTs were included. All patients were females living at high altitudes (>2500 m). In the Shamblin classification, 12 tumors were type II, and 3 were type III. Complete tumor resection was performed in all patients without pre-operative embolization. All patients had benign CBTs with a mean follow-up of 17, 73 months. In a time when the medical cost is high mainly in low-income countries such as Ecuador, further investigation should be undertaken in the form of randomized prospective trials to answer who would benefit from the pre-TAE procedure.

## INTRODUCTION

Carotid body tumors (CBTs) are rare neuroendocrine neoplasms with an incidence of 1:30 000–1:1 000 000 [1]. Most of the CBTs (~90%) are sporadic, and 10% are familial [2].

The etiology of the CBTs is associated with conditions producing chronically decreased oxygen tension such as chronic obstructive pulmonary disease and living at high altitude [3]. La Paz, Bolivia, a city 3625 m above the sea (mas) has reported a high frequency of CBTs in its population [4]. Another example that supports this hypothesis is the Tibet Plateau in China with a mean altitude of more than 3000 m [5].

Regarding the diagnosis, patients generally present with a painless, slowly enlarging lateral neck mass, accompanied by symptoms such as dysphagia or odynophagia related to the compression of the nerve structures surrounding the mass [1]. Moreover, CBTs remain challenging in intra-operative surgical resection due to the highly vascularized characteristic tumors. Therefore, they require pre-operative transarterial embolization (pre-TAE) to be removed, avoiding blood loss, reducing the operative time during surgery and decreasing the risk of perioperative complications, especially for Shamblin class II/III tumors, the CBTs classification about the carotid vessels [6].

However, recent studies have cast doubts and questioned the benefits and the utility of pre-TAE on surgery due to economic cost and have found no differences in blood loss, stroke, cranial nerve injury and length of hospital stay compared between patients with and without pre-TAE [7, 8].

Under this principle, this study aims to present our experience in patients with CBTs who underwent surgical resection without pre-TAE in a highland country, Ecuador, with >2500 mas.

## METHODS

This case series study was approved by the hospital Ethics Committee. This study was written following the STROBE guidelines for observational studies.

### Setting and participants

This single-center, case series study, was conducted at ITECC (Instituto de la Tiroides y Enfermedades de Cabeza y Cuello), a private reference center for the surgical management of CBTs in Quito, Ecuador. From July 2019 to June 2022, we included 15 patients who underwent surgery for carotid body glomus.

### Pre-operative assessment

All patients underwent thorough examinations before treatment. Image processing, such as ultrasound (US), computed tomography (CT) and/or magnetic resonance (MR), was performed for each patient as a routine preoperative examination. According to the literature, preoperative trans arterial embolization (pre-TAE) is routinely recommended for Shamblin class II and III tumors. However, none of our patients underwent pre-TAE because of the expensive costs of this procedure in our country ($18 000).

### 3D bio model reconstruction

**Figure 1:**
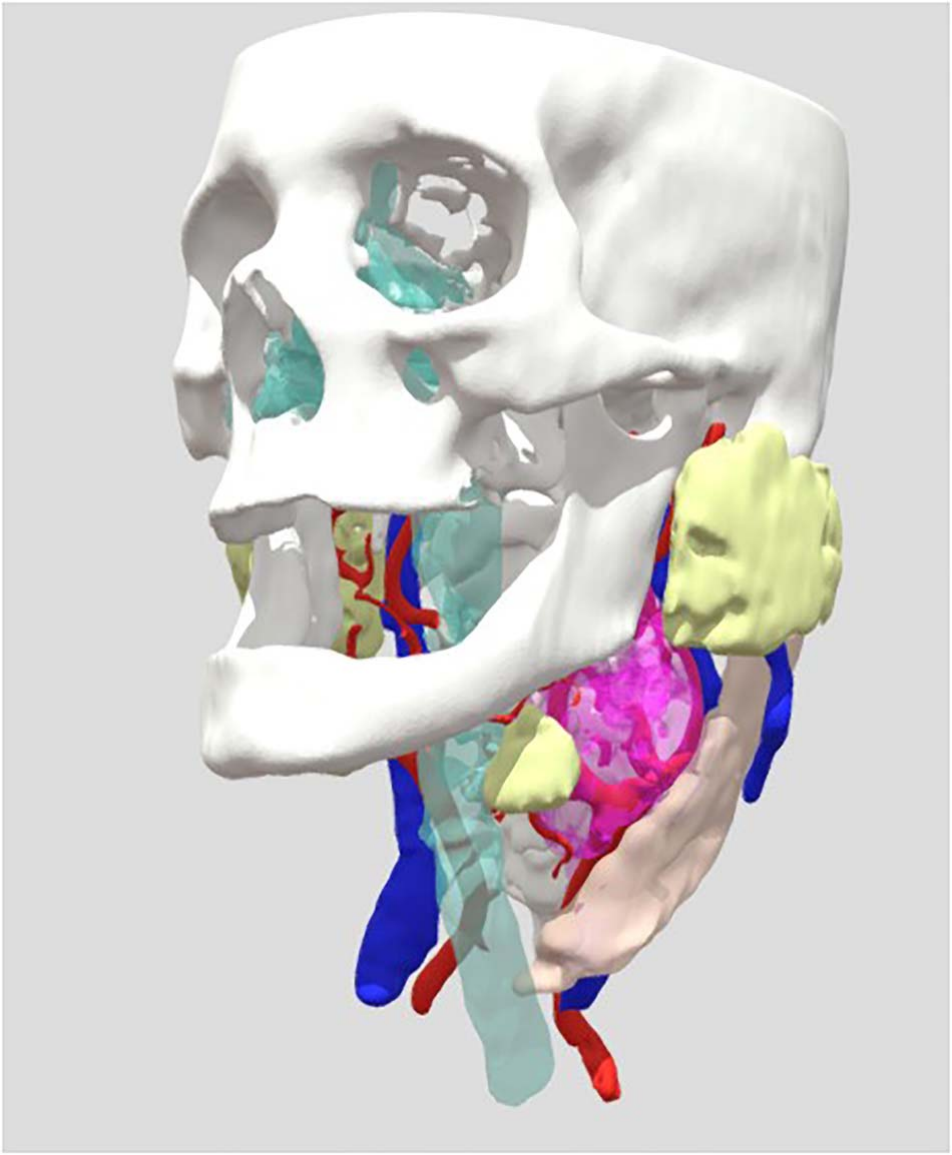
View of the reconstruction of the 3D Bio model, to observe: https://sketchfab.com/3d-models/cahuasqui-93d3db2796da46318fa9bb73802e4d01.

The 3D model reconstruction used in some patients was performed using computed tomography of the anatomical area, listed in Fig. 1. This technique allowed the surgeon to more accurately locate critical structures, anticipate intraoperative challenges and select the best surgical approach. It also improved doctor–patient communication, allowing the latter to better understand their pathology and the suggested procedure.

### Surgical technique

Briefly, after making an incision on the anterior border of the sternocleidomastoid muscle, ~4 cm under the horizontal body of the mandible with general anesthesia, subcutaneous tissue and platysma were separated and the CBT was exposed. CCA, ICA and ECA were controlled and individualized with vessel loop separators, and the common veins potentially impeding the progress of surgery were ligated using bipolar cauterization and harmonic instrumentation. The feeding vessels of the tumor were ligated meticulously. We did not need surgery to repair ICA and/or CCA in any of our patients. Finally, all resected masses underwent a histopathological examination.

### Follow-up evaluation

The mean follow-up was 17. 73 months (SD 10.89). The first follow-up was performed 1 month after treatment, then the patients were followed up by clinic visits for 12 months to 3 years.

### Statistics

Statistical analysis was performed by using the R program. Demographic data describing the presentation, pre-operative and intraoperative details and post-operative complications are reported. Descriptive statistics of the study population were calculated using arithmetic means with standard deviations for normally distributed continuous variables, median with interquartile ranges for non-normal continuous variables, and proportions for categorical variables.

## RESULTS

### Demographic characteristics

A total of 15 patients with CBTs were included from May 2019 to July 2022. All patients were females aged from 36 to 70 years old (mean age, 57 years old) and living at high altitudes (>2500 m) (Table 1).

**Table 1:** General Data

Variable^*^	Total (*n* = 15)
**Sex (*n = 15)***	
Female	15 (100%)
**Mean of age at diagnosis (*n = 15)* median (IQR)**	62 (36–70)
**Residence (*n = 15)***	
Quito	8–53, 33%
Ibarra	2–13, 33%
Cayambe	1–6, 67%
Riobamba	1–6, 67%
Cuenca	1–6, 67%
Latacunga	1–6, 67%
Peru	1–6, 67%
**Occupation (*n = 15)***	
Domestic chores	5–33, 33%
Pensionary	2–13, 33%
Labor	6–40%
S/N	2–13, 33%
**Comorbid conditions (*n = 15)***	
Obesity or Diabetes	S/N
Hypothyroidism	3–20%
Hypertension	6–40%
Other	5–33, 33%
**Family history of cancer (*n = 15)***	
Yes	5–33, 33%
No	10–66,67%
**Symptoms (*n = 15)***	
Tinnitus	2–13, 33%
Headache	2–13.33%
None	13–86.67%
**Altitude (*n = 15)***	
Greater than 2500 mas	15–100%

### The baseline features of CBTs

**Figure 2:**
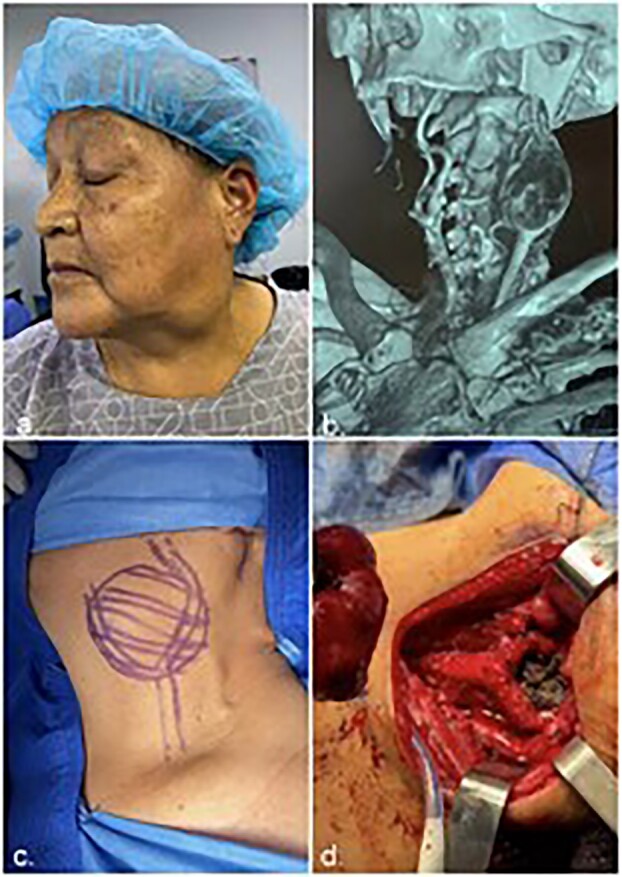
**(a)** Patient with palpable neck mass (carotid body tumor), **(b)** CT scan of a patient from the case series, **(c)**draft of Shamblin classification and **(d)** observation of CBT commitment.

All cases were unilateral (Fig. 2b), with 8 cases occurring at the left carotid bifurcation (50.90%). All cases were sporadic CBTs (100%). According to the Shamblin classification of CBTs listed in Fig. 2c, 12 tumors were Shamblin type II, and 3 were type III, resulting in a Shamblin ratio of ∼4:1. The mean tumor diameter was 3.03 cm (range: 1.5–4.5 cm) (Table 2). The most common auxiliary examination used for the diagnosis of CBTs was computed tomography (CT, 15 cases).

**Table 2:** Tumor Characteristics

Variable^*^	Total (*n* = 15)
**Laterality (*n = 15)***	
Right	7
Left	8
	
**Shamblin’s classification (n = 15)**	
Shamblin type I	0
Shamblin type II	12
Shamblin type III	3
***Tumor Size (n = 15) mean (SD)***	***3.03 cm (1.05)***
> 2 cm	13
< 2 cm	2
**Embolization in surgery**	
** Yes**	0
** No**	15
**Transfusion in surgery**	
** Yes**	0
** No**	15
**Post-surgery complications**	
** Complications < 24 h (early)**	0
** Complications > 24 h (late)**	1 (CN X dissection)
**Histology type (*n = 15)***	
Malignant tumor	0
Benign tumor	15

### The clinical manifestations of CBTs

In this study, most of the patients were asymptomatic with an incidental finding. Two patients had tinnitus and two patients reported headache (Table 1).

### The outcome of resection without pre-operative embolization

Complete tumor resection was performed in all patients without pre-operative embolization. We did not use carotid artery ligation, vessel replacement, carotid shunt, replacement materials including autogenous veins or grafts. The post-operative hospitalization was 24 hours in 93.3% of patients (*n* = 14/15).

### The complications of surgical resection

Only one patient reported a cranial nerve (CN) X resection complication due to tumor invasion (Table 2). There were no other complications (hematoma, dysphasia, seizure, dyspnea, tongue slant, hoarseness, cough, paresthesia of the neck or hypotension).

### Pathological results and follow-up

**Figure 3:**
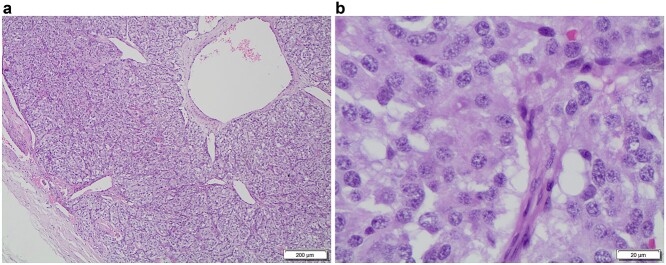
**a** (G240-22 HE 4x A): a low-power microscopic view of an encapsulated paraganglioma, note the characteristic cell nests called ‘Zellballen’, with tumor cells surrounded by thin-walled vessels; **b** (F85-22 HE 40x): this high-power view shows the tumor cells with large amounts of oncocytic cytoplasm, round to elongated nuclei with prominent nucleoli and granular chromatin.

Based upon the pathological examination, all the patients had a benign CBT with the characteristic nested pattern (Fig. 3a and b) [1] of these neuroendocrine neoplasms. There were 15 cases with completed follow-up data with a mean follow-up of 17. 73 months (SD 10.89); the overall survival rate during follow-up was 100% by Kaplan–Meier method.

## DISCUSSION

We conducted a cross-sectional analysis of all CBTs patients who underwent surgical resection without pre-TAE at a private hospital in Ecuador. This analysis revealed that all patients were females living at high altitudes. The majority were asymptomatic CBTs presenting as a unilateral mass with a mean tumor size of 3 cm and a Shamblin classification type II, and III. Despite all patients did not have pre-TAE, the surgery outcomes were successful with excellent overall survival.

CBTs are hyper vascularized tumors diagnosed mainly in women at advanced ages. Indeed, the female gender predominated, but most were between 35 and 65 years old in our study. Moreover, female patients from high altitudes more commonly had CBTs than patients from low altitudes (<1500 mas). A study from Cochabamba, Bolivia (2600 mas) reported 30 patients with a female predominance (24/30), with a mean tumor size of 3.5 cm and just two patients underwent pre-TAE [4]. Also, a recent study located in Tibet Plateau in China (3000 mas) showed 122 CBT without pre-TAE and with a maximal diameter of tumors of 3.99 cm [5]. It has been postulated that the monthly loss of blood through menstruation in women and a larger pulmonary capacity and greater enthusiasm for sports and athletic conditioning in men may allow males to escape chronic hypoxia and account for this wide gender gap [9].

These tumors grow slowly and may not cause symptoms for several years. The most common symptom is noticing a painless mass in the neck. In our study, most of the patients had an incidental finding and only two patients had tinnitus and headache. Though our results showed similar findings of CBT patients to the previous reports [10]. Similarly, our results indicated no difference between the incidence of CBT in the right and left locations. In addition, the CBTs were mainly classified as Shamblin II and III. A recent Chinese study reported a ratio of ∼1:1.7:1 in Shamblin type I, II and III, respectively [5].

Surgical resection remains the gold standard treatment option for CBTs. Pre-operative embolization is a current indication mainly in Shamblin II and III tumors to reduce tumor size, surgical bleeding and resection-related complications. However, the effectiveness of embolization is still controversial [8, 11–13]. A comparative study of patients with and without pre-TAE demonstrated no benefit of pre-operative CBTs embolization about blood loss, stroke, cranial nerve injury and length of hospital stay [13]. Based on cases of surgically resolved carotid body tumors at our institution, we have performed surgical resections without pre-operative embolization in all patients with carotid body tumors to date.

Histopathological examination is mandatory to confirm the benign nature of these tumors and to exclude the locally invasive and metastatic categories. A tumor is considered malignant only if there is metastasis to regional lymph nodes or more distant sites, such as lungs and bones [14]. The differential diagnosis includes paraganglioma-like medullary thyroid carcinoma, metastatic carcinoid tumor, hyalinizing trabecular adenoma and Hürthle cell neoplasm. Inmunostains are useful to differentiate these entities [15].

Complications after resection of carotid glomus tumors are reported in a range of 25–49% in the literature [16]. Regarding Shamblin III tumors, the most common complication is nerve injury between a range of 20 and 50% [17]. Our study did not report major complications such as vascular, nervous or mortality complications. One patient had a dissection of CNX due to tumor invasion. Moreover, the literature reported a malignancy rate of ~5% [18]. In our study, we had no report of malignancy or lymph node metastasis in any patient.

## CONCLUSION

In a time where medical cost and surgical outcomes are so highly scrutinized, mainly in low-income countries such as Ecuador, further investigation should be undertaken in the form of randomized prospective trials to answer who would benefit from the pre-TAE procedure.
